# In Vitro Anti-*Aspergillus* Activity and Antifungal Interactions of Monoclonal Antibodies 1D2 and 4E4

**DOI:** 10.3390/jof12070523

**Published:** 2026-07-16

**Authors:** Xihua Lian, Amy Scott-Thomas, John G. Lewis, Madhav Bhatia, Stephen T. Chambers

**Affiliations:** 1 Department of Ultrasound Medicine, Second Clinical Medical School, Fujian Medical University, Quanzhou 362000, China; xihua.lian@fjmu.edu.cn; 2 Department of Pathology and Biomedical Science, University of Otago, Christchurch 8011, New Zealand; amy.scott-thomas@otago.ac.nz (A.S.-T.); john.lewis@otago.ac.nz (J.G.L.)

**Keywords:** monoclonal antibody, 1D2, 4E4, *Aspergillus* infection, invasive aspergillosis, antifungal agents

## Abstract

Objective: To determine the in vitro anti-*Aspergillus* activity of monoclonal antibodies (mAb) 1D2 and 4E4 alone and in combination with voriconazole, posaconazole, amphotericin B and caspofungin and to assess whether selected antimicrobial agents interfere with 1D2/4E4 binding to *Aspergillus* antigens. Methods: The in vitro antifungal activity of 1D2, 4E4 and all antifungal drugs was determined in a microplate-based assay. The anti-*Aspergillus* activity of mAb 1D2 and 4E4 in vitro alone and in combination with voriconazole, posaconazole, amphotericin B and caspofungin was determined using a checkerboard assay. Finally, a competitive ELISA was used to assess whether selected antifungal and antibacterial agents interfered with the binding of 1D2 and 4E4 to immobilised *Aspergillus* antigens. Results: The in vitro interactions of 1D2 with voriconazole, posaconazole, amphotericin B and caspofungin against four different *Aspergillus* species were all additive, with a fractional inhibitory concentration index (FICI) between 0.5 and 1. Similarly, the in vitro interactions of 4E4 with these same four antifungals against *Aspergillus fumigatus* (*A. fumigatus*) were all additive. An additive interaction was also detected for 4E4 and caspofungin against all four *Aspergillus* species tested. However, no interaction was found for 4E4 combined with voriconazole, posaconazole and amphotericin B against *Aspergillus flavus* (*A. flavus*), *Aspergillus niger* (*A. niger*) and *Aspergillus terreus* (*A. terreus*), with an FICI between 1 and 4. Moreover, none of the tested antifungal or antibacterial agents significantly interfered with 1D2/4E4 binding to *Aspergillus* antigens in the competitive ELISA. Conclusions: In this study, 1D2 and 4E4 showed in vitro growth-inhibitory activity against the tested *Aspergillus* isolates. Specifically, 1D2 showed additive interactions with the tested antifungal agents across all tested species, whereas 4E4 showed a more species- and drug-dependent interaction profile. The tested antimicrobial agents did not measurably interfere with 1D2/4E4 binding under the competitive ELISA conditions examined. Overall, these findings provide preliminary in vitro evidence supporting the further evaluation of 1D2 and 4E4.

## 1. Introduction

The incidence of *Aspergillus* infections has increased among immunocompromised populations due to the increasingly frequent usage of immunosuppressive regimens and anticancer therapies [1,2]. Current guideline appraisals indicate that voriconazole and isavuconazole are commonly recommended as first-line therapies for invasive aspergillosis, while posaconazole, amphotericin B formulations and echinocandins are used in selected prophylactic, alternative, salvage or combination settings [3,4,5,6]. Although efforts have been made to explore new antifungal agents for treating invasive aspergillosis (IA), the high toxicity, antifungal resistance and limited activity spectra of currently available drugs are of high concern. Azole-resistant *A. fumigatus* has been increasingly reported after long-term clinical azole exposure and through environmental selection associated with agricultural azole fungicides [7,8,9]. As such, there is an urgent need to pursue new anti-*Aspergillus* strategies with improved specificity, reduced toxicity and broader activity against clinically relevant *Aspergillus* species.

We have previously generated monoclonal antibodies (mAbs) 1D2 and 4E4 against *Aspergillus* cell wall glycoprotein antigens [10,11]. Both antibodies recognise *Aspergillus* cell wall-associated antigens and have been evaluated as potential diagnostic antibodies for detecting *Aspergillus* antigens during early invasive aspergillosis [11]. In our previous in vitro study, 1D2 inhibited several early fungal developmental processes, including conidial swelling, germination and hyphal growth, and reduced the metabolic activity of *Aspergillus fumigatus* (*A. fumigatus*) conidia [10]. Growth inhibition was maximal at 50 μg/mL, and 1D2 impaired swelling and germination even when added up to seven hours after inoculation. Moreover, 4E4 has shown similar in vitro effects on *Aspergillus* species to 1D2.

Based on these findings, we hypothesised that 1D2 and 4E4 may have therapeutic potential for IA. In this study, therefore, we determined the anti-*Aspergillus* activity of 1D2 and 4E4, both alone and in combination with commonly used antifungal agents. In addition, patients at risk of IA frequently receive broad-spectrum antibacterial and antifungal therapy, and false-positive *Aspergillus* antigen results have been reported in association with antimicrobial agents, intravenous fluids and other clinical factors [12,13,14,15]. Some β-lactam agents, such as piperacillin–tazobactam, may also contain fungal antigens derived from the fermentation manufacturing process, which can contribute to galactomannan assay reactivity [16]. We therefore used a competitive ELISA to assess whether selected antibacterial and antifungal agents interfered with the binding of 1D2 and 4E4 to *Aspergillus* antigens.

## 2. Materials and Methods

### 2.1. Fungal Strains and Growth Conditions

*A. fumigatus* (AF293) and *Aspergillus flavus* (*A. flavus*, NRRL 3357) were obtained from the American Type Culture Collection (ATCC). In addition, clinical isolates of *A. fumigatus*, *Aspergillus niger* (*A. niger*) and *Aspergillus terreus* (*A. terreus*) were provided by Canterbury Health Laboratories, Christchurch, New Zealand. All fungal strains were cultured on Sabouraud dextrose agar (SDA) plates or in Sabouraud dextrose (SD) liquid media at 37 °C.

### 2.2. Preparation of Aspergillus Conidia Suspension

*Aspergillus* conidial suspensions were prepared as previously described, with small modifications [17]. All *Aspergillus* species were cultured on SDA plates for 5–7 days to acquire conidia. The plates were washed with phosphate-buffered saline containing 0.1% Tween 20 (PBST) to obtain spores and the collected conidial suspensions filtered by 8-layer Miracloth (Merck, Darmstadt, Germany) and washed with PBST three times. Finally, the conidial suspension was adjusted to 5 × 10^6^ conidia/mL and diluted 100-fold in RPMI-1640-3-(N-morpholino) propanesulfonic acid (RPMI-1640-MOPS) media to a final concentration of 5 × 10^4^ conidia/mL.

### 2.3. Preparation of Antifungals and Antibiotics

Voriconazole, posaconazole, amphotericin B and caspofungin (Sigma-Aldrich, Auckland, New Zealand) were solubilised in dimethyl sulfoxide (DMSO) and diluted in RPMI-1640-MOPS media to specific concentrations according to the assay.

Piperacillin, ceftazidime, meropenem, ciprofloxacin, vancomycin and gentamicin (Sigma-Aldrich, Auckland, New Zealand) were tested in this study. All were dissolved in distilled water and diluted in PBS to specific concentrations according to the assay.

### 2.4. In Vitro Growth-Inhibitory Activity of Individual Agents

The in vitro growth-inhibitory activity of 1D2, 4E4 and the antifungal drugs was assessed using a broth microdilution-based assay adapted from the European Committee on Antimicrobial Susceptibility Testing (EUCAST) method [18], with some modifications. Broth dilution was used to determine the minimum inhibitory concentrations (MICs) of antifungal agents for conidia-forming moulds. The drug stock was diluted in RPMI-1640-MOPS media to obtain two times the final concentration. A series of two-fold dilutions of each drug, two times the final concentration, was added to columns 1 to 12 (100 μL/well) of a 96-well plate, in triplicate, followed by the addition of 100 μL/well of conidial inoculum to each well. An isotype-matched irrelevant IgM mAb 6B10 was included at the same concentration range as 1D2 and 4E4 as an antibody control to assess non-specific effects of IgM or antibody protein on *Aspergillus* growth. Growth control wells contained conidia without antifungal drugs, and blank controls contained culture media only. The microtiter plate was incubated without shaking at 37 °C for 48 h. Absorbance was read by a spectrophotometer at 405 nm. The growth inhibition percentage was calculated as follows:

$$\text{Growth inhibition percentage}=\frac{1-(\text{A405 sample}-\text{A405 blank})}{\left(\text{A405 growth}-\text{A405 blank}\right)}\times 100$$

For voriconazole, posaconazole and amphotericin, the minimum inhibitory concentration (MIC) was defined as the lowest concentration of the agent that caused no visible growth (visual observation) or more than a 95% growth reduction (spectrophotometric method) in the microorganism when compared to the drug-free growth controls. In addition, the MIC endpoint was determined by both visual observation and spectrophotometry [18]. The minimum effective concentration (MEC) was defined for caspofungin as the lowest concentration that led to the growth of small, rounded, compact hyphal forms, compared to normal hyphal growth typically observed in the control wells [18]. For monoclonal antibodies, an MIC-like growth-inhibitory endpoint was used operationally and was defined as the lowest antibody concentration producing no visible growth or ≥95% growth reduction under the assay conditions. This endpoint was used to describe antibody-associated growth inhibition and was not interpreted as a conventional antifungal MIC.

### 2.5. In Vitro Checkerboard Assay of 1D2/4E4 and Antifungals Against Aspergillus

The checkerboard technique was based on the EUCAST broth microdilution method for conidia-forming moulds, with modifications for antifungal combination testing [18,19,20]. Antifungal agents were diluted in RPMI-1640-MOPS media at four times the final concentration. The working concentration ranges of the antifungal drugs were

• 0.06 to 64 μg/mL for voriconazole, posaconazole and amphotericin B;
• 0.008 to 4 μg/mL for caspofungin.

**Figure 1:**
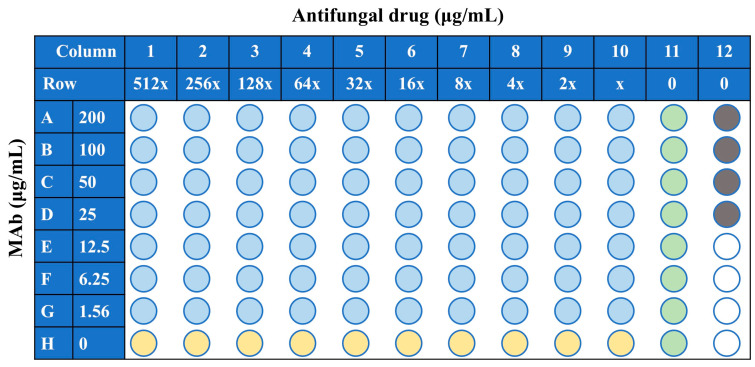
Checkerboard plate set up for the analysis of mAb and antifungal drugs. A 96-well microplate was used to test the in vitro titration with mAb 1D2 and 4E4 alongside antifungal drugs against *Aspergillus*. The same layout was used for parallel assays with the isotype-matched IgM control antibody 6B10. x: four times the lowest final concentration; blue wells: sample wells; grey wells: growth controls; yellow wells: antifungal drug controls; green wells: mAb controls; white wells: blank controls.

A sterile 96-well microplate was set up to complete the in vitro checkerboard titration with mAbs 1D2/4E4 and antifungal drugs against *Aspergillus*. As shown in Figure 1, the initial template concentrations of the monoclonal antibodies ranged from 0 to 200 μg/mL. A series of dilutions of each drug with four times the final concentration was added to columns 1 to 10 (50 μL/well) in triplicate. Columns 11 and 12 acted as control columns containing culture media only. A series of dilutions of mAbs was added to rows A to G (50 μL/well). Parallel checkerboard assays were performed using the isotype-matched IgM control antibody 6B10 at the same concentration range to control for non-specific antibody-related effects during combination testing. Culture medium alone was added to row H. Subsequently, 100 μL/well of conidial inoculum was added to each well. Growth control wells contained conidia without antifungal drugs or antibody. Drug control wells contained either only antifungal drugs or mAbs. Thus, following the addition of mAbs (50 μL/well), antifungal drugs (50 μL/well) and the conidial inoculum (100 μL/well), the final concentration of the mAbs and antifungal drugs was 1/4 of that shown in the template in Figure 1. The microtiter plate was incubated without shaking at 37 °C for 48 h. The absorbance of the plate was read by a spectrophotometer (Thermo Fisher Scientific, Auckland, New Zealand) at 405 nm.

The growth inhibition percentage was calculated as follows:

$$\text{Growth inhibition percentage}=\frac{1-(\text{A405 sample}-\text{A405 blank})}{\left(\text{A405 growth}-\text{A405 blank}\right)}\times 100$$

The minimum inhibitory concentrations (MICs) and minimum effective concentration (MEC) were used to evaluate both the individual and combined effects of antifungal agents and mAb 1D2/4E4, where the MIC endpoint was determined by both visual inspection and spectrophotometry [18]. The fractional inhibitory concentration (FIC) of each drug was calculated as follows:

$$\text{FIC A}=\frac{MIC(\text{A in the presence of B})}{MIC(\text{A alone})}\times 100$$

To assess the interaction between the antifungal agents and mAb 1D2/4E4, the FIC index (FICI) was calculated as follows:

$$\begin{array}{l} FICI=\text{FIC A}+\text{FIC B}\\ \;=[MIC(\text{A in the presence of B})/MIC(\text{A alone})]\\ \;+[MIC(\text{B in the presence of A})/MIC(\text{B alone})] \end{array}$$

The interaction between two agents was defined using the FICI as follows: FICI ≤ 0.5 shows a synergistic relationship, 0.5 < FICI ≤ 1 shows an additive relationship, 1 < FICI ≤ 4 shows no interaction (indifference), and > 4 shows an antagonistic relationship [19,20,21].

### 2.6. Competitive ELISA for Assessing Antimicrobial Interference with 1D2/4E4 Binding

A competitive ELISA was used to assess whether the selected antibacterial and antifungal agents interfered with the binding of 1D2 and 4E4 to immobilised *A. fumigatus* cell wall fragments (CWFs). Briefly, *A. fumigatus* CWFs were coated onto 96-well microtitre plates at 176 µg/mL. After washing and blocking, antibacterial or antifungal agents were prepared at predefined starting concentrations and serially diluted from 1:1 to 1:64 before being added to the wells. The starting concentrations before serial dilution were 2100 µg/mL for piperacillin, 300 µg/mL for meropenem, 700 µg/mL for ceftazidime, 50 µg/mL for ciprofloxacin, 400 µg/mL for vancomycin and 80 µg/mL for gentamicin. For antifungal agents, the starting concentrations before serial dilution were 2.0 mg/mL for caspofungin and 16 mg/mL for amphotericin B, voriconazole and posaconazole. Each antimicrobial dilution was added at 50 μL/well, immediately followed by 50 μL/well of 1D2 or 4E4 at 5 μg/mL. Because each antimicrobial dilution was mixed with an equal volume of antibody solution, the final in-well concentrations were half of those of the corresponding prepared dilutions. The plates were incubated for one hour at room temperature. Wells coated with *A. fumigatus* CWFs and incubated with antibody in the absence of antimicrobial agents served as positive binding controls, whereas PBS-coated wells served as negative controls. Assay signals were measured using a spectrophotometer.

### 2.7. Statistical Analysis

All statistical analyses were completed using the SPSS software (version 21.0, IBM Corp., New York, NY, USA) and GraphPad software (version 9, Prism, San Diego, CA, USA). Unless otherwise specified, all data were derived from three independent experiments, and continuous and normally distributed data values were presented as [mean ± standard deviation]. To detect a difference between three or more groups, a one-way ANOVA with the post hoc Dunnett T3/Tukey’s multiple-comparisons test or the Kruskal–Wallis test with the post hoc Dunn’s multiple-comparisons test was performed. A *p* value < 0.05 was considered statistically significant.

## 3. Results

### 3.1. In Vitro Growth-Inhibitory Activity of mAb 1D2/4E4 and Individual Agents

**Figure 2:**
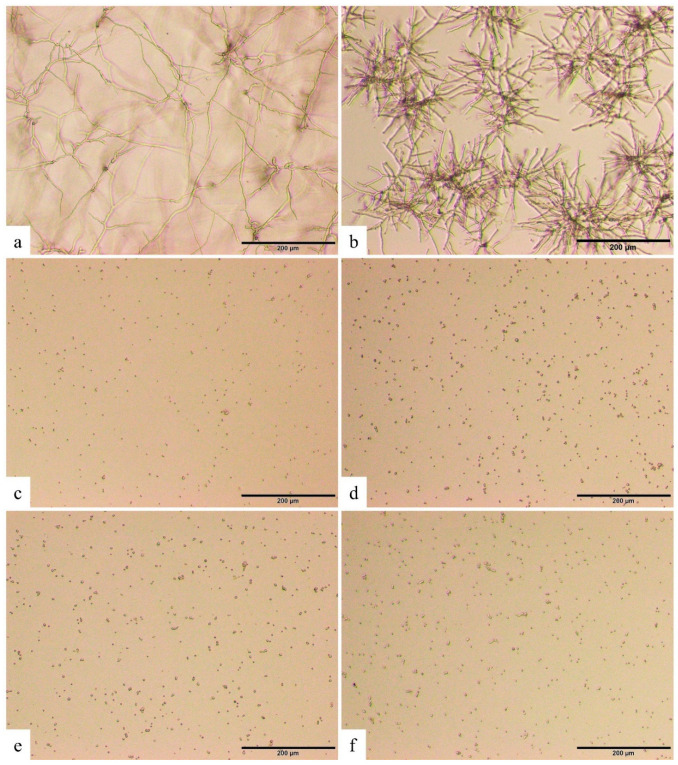
*A. fumigatus* growth morphology after incubation with antifungal drugs and 1D2 or 4E4 at minimum inhibitory concentration (MIC) or minimum effective concentration (MEC). *A. fumigatus* conidia formed long hyphae in the growth control (**a**). Caspofungin (MEC: 0.25 µg/mL) caused the growth of small, rounded and compact hyphae (**b**) compared to the growth control (**a**). Voriconazole (MIC: 1.0 μg/mL) (**c**), posaconazole (MIC: 0.5 μg/mL) (**d**) and amphotericin B (MIC: 0.5 μg/mL) (**e**) caused no visible hyphae. In addition, mAb 1D2/4E4 at an MIC-like growth-inhibitory endpoint of 12.5 µg/mL (**f**) resulted in no visible hyphal growth. This assay was performed in triplicate. Scale bars represent 200 μm. MIC: minimum inhibitory concentration; MEC: minimum effective concentration.

The tested isolates of *A. fumigatus*, *A. flavus*, *A. niger* and *A. terreus* showed growth inhibition by mAb 1D2 and 4E4, as well as by voriconazole, posaconazole, amphotericin B and caspofungin, under the in vitro conditions used. The in vitro growth-inhibitory activity of each antibody and each drug against various *Aspergillus* species is summarised in Table 1. Figure 2 shows that *A. fumigatus* conidia formed long hyphae with septa in the growth control groups (Figure 2a). An MEC of 0.25 μg/mL for caspofungin led to the growth of small, rounded and compact hyphae in comparison to hyphae observed in the growth control wells (Figure 2b). The MICs were 1.0 μg/mL, 0.5 μg/mL and 0.5 μg/mL for voriconazole, posaconazole and amphotericin B, respectively, and caused no visible hyphal growth or reduced hyphae growth by more than 95% for *A. fumigatus* (Figure 2c–e). No visible hyphal growth was observed for 1D2 or 4E4 at an MIC-like growth-inhibitory endpoint of 12.5 μg/mL (Figure 2f). In contrast, the isotype antibody 6B10 did not produce comparable growth inhibition at matched concentrations, indicating that the inhibitory effects observed with 1D2 and 4E4 were unlikely to be attributable to non-specific IgM-related effects.

### 3.2. Antifungal Activity of 1D2 and 4E4 in Combination with Antifungal Agents Against Different Aspergillus Species

**Figure 3:**
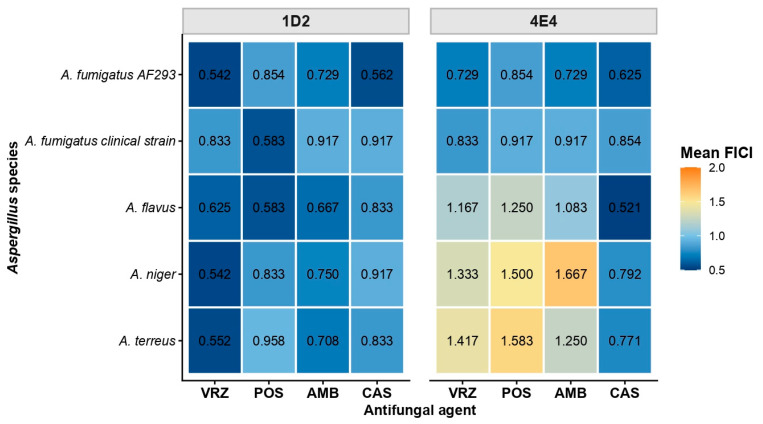
Global interaction profile of 1D2 and 4E4 with antifungal agents against *Aspergillus* species. Heatmap showing mean fractional inhibitory concentration index (FICI) values from three independent checkerboard assays. The left and right panels show 1D2 and 4E4, respectively. Lower FICI values, shown in darker blue, indicate stronger interactions, whereas higher values, shown in yellow-to-orange tones, indicate reduced or absent interactions. FICI was interpreted as synergy (≤0.5), additive interaction (>0.5 to ≤1), no interaction (>1 to ≤4) or antagonism (>4). VRZ, voriconazole; POS, posaconazole; AMB, amphotericin B; CAS, caspofungin.

The antifungal activity of 1D2 and 4E4 against different *Aspergillus* species in combination with antifungals is shown in Table 2 and Table 3. Table 2 shows that all antifungal drugs had an additive effect with 1D2, with FICI values between 0.5 and 1.0. Similarly, Table 3 indicates that the in vitro interactions of 4E4 with voriconazole, posaconazole, amphotericin B and caspofungin against *A. fumigatus* were all additive. An additive interaction was also detected for caspofungin and 4E4 against all *Aspergillus* species; however, no interaction was found for 4E4 with voriconazole, posaconazole and amphotericin B against *A. flavus*, *A. niger* and *A. terreus*, with an FICI value between 1.0 and 4.0. To provide an overall visual summary of the interaction patterns, the mean FICI values for all antibody–antifungal combinations were further displayed as a global heatmap (Figure 3). This visualisation highlighted the broad additive interaction of 1D2 with the tested antifungal agents and the more species-dependent interaction profile of 4E4.

### 3.3. Assessment of Antibacterial Drug Interference with 1D2/4E4 Binding

**Figure 4:**
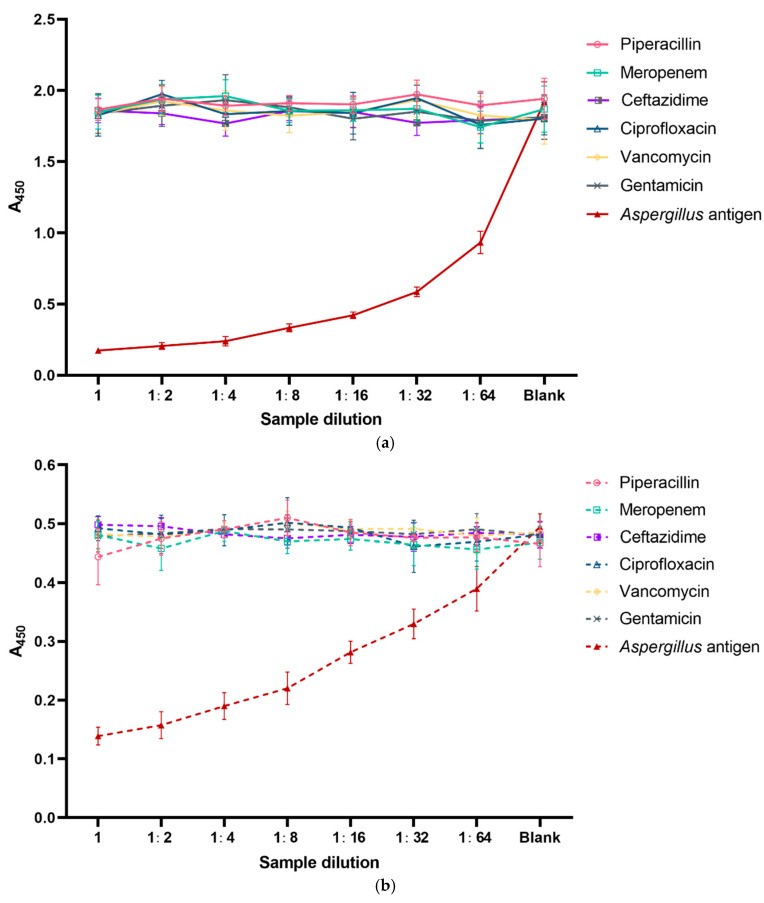
Assessment of antibacterial drug interference with 1D2/4E4 binding to *A. fumigatus* antigens. Competitive ELISA was used to assess whether antibacterial agents interfered with the binding of 1D2 (**a**) and 4E4 (**b**) to immobilised *A. fumigatus* cell wall antigens. Piperacillin, meropenem, ceftazidime, ciprofloxacin, vancomycin and gentamicin did not significantly reduce the binding signals of 1D2 or 4E4 compared with the *A. fumigatus* antigen control group (176 µg/mL). The assay was repeated three times. Data are presented as mean ± SD.

Piperacillin, meropenem, ceftazidime, ciprofloxacin, vancomycin and gentamicin did not significantly reduce the binding signal of 1D2 to immobilised *A. fumigatus* antigens compared with the *A. fumigatus* antigen control group (176 µg/mL) (Figure 4a). Similarly, none of the tested antibacterial agents affected the binding of 4E4 to immobilised *A. fumigatus* antigens (Figure 4b). These findings suggest that the tested antibacterial agents did not interfere with 1D2/4E4 antigen binding under the conditions used in this competitive ELISA.

### 3.4. Assessment of Antifungal Drug Interference with 1D2/4E4 Binding

**Figure 5:**
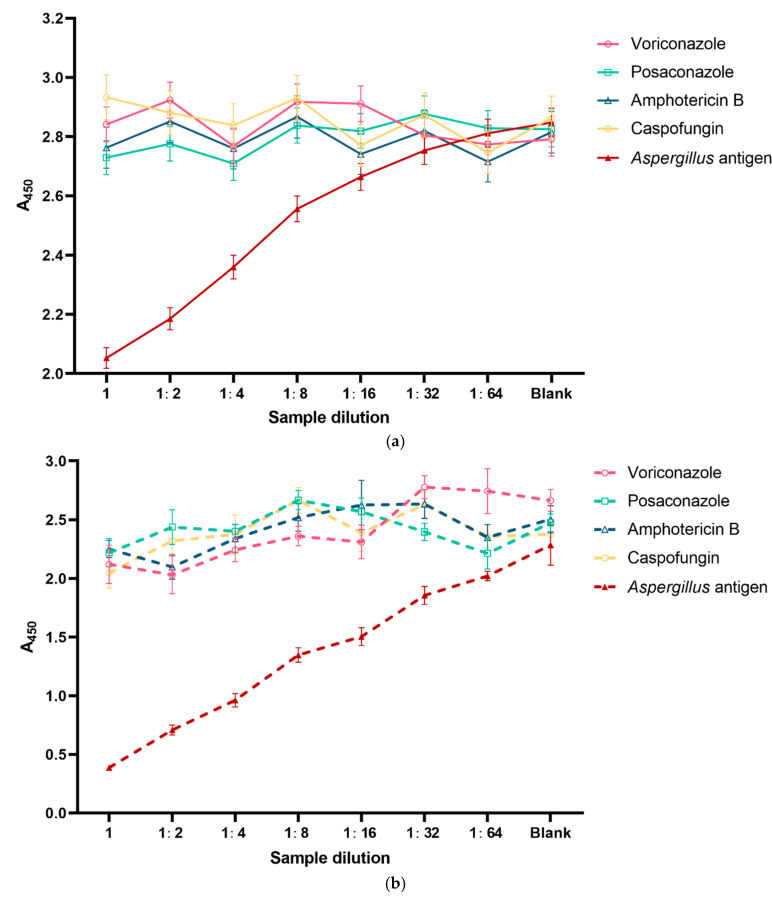
Assessment of antifungal drug interference with 1D2/4E4 binding to *A. fumigatus* antigens. Competitive ELISA was used to assess whether antifungal agents interfered with the binding of 1D2 (**a**) and 4E4 (**b**) to immobilised *A. fumigatus* cell wall antigens. Voriconazole, posaconazole, amphotericin B and caspofungin did not significantly reduce the binding signals of either antibody compared with the *A. fumigatus* antigen control group (176 µg/mL). The assay was repeated three times. Data are presented as mean ± SD.

Voriconazole, posaconazole, amphotericin B and caspofungin did not significantly reduce the binding signal of 1D2 to immobilised *A. fumigatus* antigens compared with the antigen control group (176 µg/mL). Similarly, none of the tested antifungal agents affected the binding of 4E4 to immobilised *A. fumigatus* antigens (Figure 5). These results indicate that the tested antifungal agents did not interfere with 1D2/4E4 binding to immobilised *A. fumigatus* antigens under the assay conditions examined.

## 4. Discussion

In this study, the clinically relevant *Aspergillus* isolates showed growth inhibition by mAbs 1D2 and 4E4, as well as by the conventional antifungal agents tested. We also determined the minimum inhibitory concentrations (MICs) of voriconazole, posaconazole and amphotericin B and the minimum effective concentration (MEC) of caspofungin for each tested isolate. The in vitro interactions between 1D2 and voriconazole, posaconazole, amphotericin B or caspofungin were additive across the *Aspergillus* isolates. Similarly, 4E4 showed additive interactions with the four antifungal agents against *A. fumigatus* and with caspofungin against all the isolates tested. In contrast, no interaction was observed between 4E4 and voriconazole, posaconazole or amphotericin B against *A. flavus*, *A. niger* and *A. terreus*. Moreover, the antifungal and antibacterial agents did not significantly interfere with 1D2/4E4 binding to *Aspergillus* antigens in the competitive ELISA.

Azoles, polyenes and echinocandins represent the major antifungal classes used for the treatment or management of *Aspergillus* infection [3,4,5]. We therefore selected voriconazole and posaconazole to represent triazole agents, amphotericin B to represent polyenes and caspofungin to represent echinocandins. This selection allowed us to evaluate whether 1D2 and 4E4 interact with antifungal agents that act through distinct mechanisms, including the inhibition of ergosterol biosynthesis, disruption of membrane integrity and interference with fungal cell wall synthesis [22,23,24]. The concentration ranges of each antifungal agent utilised here were wider than those previously reported and ranged from 0.016 to 16 μg/mL for voriconazole, posaconazole and amphotericin B [25] and 0.015 to 8 µg/mL for caspofungin [26]. These initial growth inhibition assays established the concentration ranges and MIC/MEC or MIC-like endpoints used for the subsequent checkerboard combination experiments.

An MIC is usually applied to assess the effectiveness of an antifungal agent; the definition of the MIC endpoint is debatable if different growth reduction percentages are used, and different conclusions can be reached [27]. Here, we defined the MIC as the lowest concentration of voriconazole, posaconazole and amphotericin B that stopped growth by visual observation or caused a 95% growth reduction in *Aspergillus* when assessed by spectrophotometry [28]. Serrano-Lobo et al. reported that the determination of azole and amphotericin B MIC endpoints against *A. fumigatus* species by spectrophotometry showed high agreement with the visual endpoint observation method [28]. In this study, the MIC endpoints of azoles and amphotericin were determined by both visual observation and spectrophotometry, making the obtained MICs more reliable. For 1D2 and 4E4, however, the endpoint was interpreted as an MIC-like growth-inhibitory endpoint rather than a conventional antifungal MIC. Because antibody effects were assessed by absorbance and morphology rather than CFU recovery, the findings should not be interpreted as direct evidence of fungicidal killing.

The determination of the in vitro MIC of caspofungin still remains unclear and less practical [29]. This is because the MIC of caspofungin is typically higher than the maximum plasma concentration, which is around 9 μg/mL in an adult receiving a 50 mg maintenance daily dose after a caspofungin 70 mg loading dose on day one [30] and about 17.5 μg/mL in children receiving a 70 mg/m^2^ loading dose followed by a 50 mg/m^2^ maintenance dose [31]. As such, a morphological change-dependent concentration, the minimum effective concentration (MEC), was utilised to assess the lowest concentration of caspofungin that caused characteristic *Aspergillus* hyphal changes compared to the growth control hyphae by EUCAST in vitro [18]. Recent XTT colorimetric assays have shown promise in determining the MEC endpoint for echinocandins [18,32]. The present study did not specifically assess the caspofungin paradoxical effect when combined with 1D2 or 4E4. Although no antagonism was observed in the checkerboard assays, paradoxical growth requires a dedicated experimental design using appropriate high-concentration caspofungin exposure, morphological assessment and growth recovery analysis. This will be important to evaluate in future studies, particularly in azole-resistant *Aspergillus* isolates.

The checkerboard method was used to test the interactions of 1D2 and 4E4, individually, with each antifungal agent against different *Aspergillus* species. Importantly, no antagonism was observed in any tested antibody–antifungal combination. This suggests that antibody binding did not measurably impair the growth-inhibitory activity of the tested antifungal agents under the in vitro conditions used, although direct effects on drug penetration or target engagement were not assessed. The additive profile of 1D2, together with the more selective additive activity of 4E4, suggests that these antibodies may be compatible with established antifungal classes in vitro. In this setting, an additive interaction indicates enhanced combined activity without meeting the threshold for synergy, whereas no interaction indicates no measurable enhancement in antifungal activity under the checkerboard conditions used, but also no evidence of antagonism.

This compatibility could be explained by complementary effects between antibody-mediated interference with fungal surface structures and the distinct mechanisms of conventional antifungal agents, including the inhibition of ergosterol biosynthesis, disruption of membrane integrity and interference with fungal cell wall synthesis [22,23]. The fungal cell wall is a dynamic surface structure involved in host recognition, antifungal stress responses and drug susceptibility, and changes in cell wall architecture may influence the activity of antifungal agents [33,34]. The species-dependent interaction profile of 4E4 may reflect differences in epitope abundance, glycoprotein composition or the accessibility of cell wall antigens among *Aspergillus* species. These differences may also be related to species-specific cell wall organisation and virulence-associated features. Therefore, one possible hypothesis is that the binding of 1D2 or 4E4 to *Aspergillus* cell wall antigens could alter fungal surface accessibility, the cell wall architecture or early hyphal development, thereby contributing to the additive interactions observed with selected antifungal agents. However, the precise glycoprotein targets recognised by 1D2 and 4E4 have not yet been fully defined. Therefore, the relationship between antibody binding specificity, interspecies differences in cell wall antigen expression and antifungal interaction patterns remains to be determined. This interpretation remains speculative and requires further mechanistic validation. Antibody-mediated effects during infection may also involve host-dependent mechanisms, but these were outside the scope of the present in vitro study and were not assessed here.

Nevertheless, the observed additive effects may warrant further evaluation as potential adjunctive anti-*Aspergillus* candidates rather than as replacements for established antifungal therapy. This interpretation is also consistent with broader evidence that antibodies against fungal antigens can modify host–fungal interactions and may have therapeutic potential in experimental fungal infections [35,36]. Given their antigen specificity and preliminary in vitro activity, further studies are warranted to evaluate their relevance in more complex experimental systems, including resistant isolates and in vivo models of invasive aspergillosis. Importantly, the additive interactions observed in vitro should not be interpreted as evidence of in vivo therapeutic efficacy or clinical benefit.

Drug-related assay interference is an important consideration for antibody-based *Aspergillus* antigen detection assays [12,13,14,15,37]. Given that mAbs 1D2 and 4E4 have also been evaluated as potential diagnostic antibodies for IA [], it was important to determine whether commonly used antimicrobial agents could interfere with the binding of these antibodies to *Aspergillus* antigens. In the present study, a competitive ELISA was used to assess whether selected antifungal and antibacterial agents, including voriconazole, posaconazole, amphotericin B, caspofungin, piperacillin, ceftazidime, meropenem, ciprofloxacin, vancomycin and gentamicin, interfered with 1D2/4E4 binding to immobilised *A. fumigatus* cell wall antigens. No measurable reduction in antibody binding was observed in the presence of any of the tested agents under the assay conditions used. These findings suggest that the tested antimicrobial agents are unlikely to directly interfere with 1D2/4E4 antigen binding in this in vitro system. However, this experiment used immobilised *A. fumigatus* cell wall antigens and did not include clinical matrices such as serum, bronchoalveolar lavage fluid or urine. Therefore, these results should not be interpreted as excluding false-positive reactivity or assay interference in clinical specimens. Further validation using clinical samples from patients receiving these antimicrobial agents is required before conclusions can be drawn regarding clinical diagnostic performance [38].

This study has several limitations. First, the experiments were performed in vitro and did not evaluate the therapeutic efficacy of 1D2 or 4E4 in an animal model of IA. Host-dependent antibody mechanisms during infection were also not assessed in this study. Second, only a limited number of *Aspergillus* isolates were tested, and azole-resistant isolates were not included. Third, although 6B10 was used as an isotype-matched IgM control, the mechanisms underlying the additive interactions between the antibodies and antifungal agents remain undefined. In addition, FICI-based in vitro interactions indicate compatibility under defined assay conditions but may not directly predict in vivo therapeutic synergy or clinical efficacy. The caspofungin paradoxical effect was also not specifically assessed in the present study and should be examined in future antibody–echinocandin combination experiments. Finally, the competitive ELISA experiments assessed drug interference under defined in vitro conditions and should be further validated using clinical specimens.

## 5. Conclusions

In conclusion, 1D2 and 4E4 showed in vitro growth-inhibitory activity against the tested clinically relevant *Aspergillus* isolates. In addition, mAb 1D2 showed additive interactions with voriconazole, posaconazole, amphotericin B and caspofungin across all tested *Aspergillus* species in vitro. Similar additive activity was detected for mAb 4E4, although no interaction was found for 4E4 with voriconazole, posaconazole or amphotericin B against *A. flavus*, *A. niger* and *A. terreus*. Moreover, the tested antifungal and antibacterial agents did not significantly interfere with the binding of 1D2 or 4E4 to *Aspergillus* antigens in the competitive ELISA. Overall, these findings should be interpreted as preliminary in vitro evidence supporting further mechanistic, diagnostic and in vivo evaluations of 1D2 and 4E4. They do not establish therapeutic efficacy or clinical diagnostic performance.

## Figures and Tables

**Table 1:** In vitro growth-inhibitory activity of antifungal drugs and 1D2/4E4 against *Aspergillus* species.

Species	Concentration		Antifungal Drug				
	(μg/mL)	MAb 4E4	MAb 1D2	Voriconazole	Posaconazole	Amphotericin B	Caspofungin
*A. fumigatus* (AF293)	Range	0.78–50	0.78–50	0.0625–128	0.0625–128	0.0625–128	0.0078–16
	MIC/MEC	12.5	12.5	1	0.5	0.5	0.25
*A. fumigatus* (clinical strain)	Range	0.78–50	0.78–50	0.0078–16	0.0078–16	0.0156–32	0.0078–16
	MIC/MEC	6.25	6.25	0.25	0.25	0.5	0.5
*A. flavus*	Range	0.78–50	0.78–50	0.0078–16	0.0078–16	0.0156–32	0.0078–16
	MIC/MEC	12.5	12.5	0.125	0.25	0.5	0.25
*A. niger*	Range	0.78–50	0.78–50	0.0625–128	0.0156–32	0.0156–32	0.0078–16
	MIC/MEC	12.5	3.125	1	0.5	0.25	0.25
*A. terreus*	Range	0.78–50	0.78–50	0.0156–32	0.0078–16	0.0625–128	0.0078–16
	MIC/MEC	25	1.5625	0.25	0.125	1	0.5

Note: This assay was performed in triplicate. MIC: minimum inhibitory concentration; MEC: minimum effective concentration. *A. fumigatus*: *Aspergillus fumigatus*; *A. flavus*: *Aspergillus flavus*; *A. niger*: *Aspergillus niger*; *A. terreus*: *Aspergillus terreus*. For monoclonal antibodies, the values indicate MIC-like growth-inhibitory endpoints rather than conventional antifungal MICs.

**Table 2:** The antifungal activity of 1D2 in combination with antifungal agents against different *Aspergillus* species.

Species	FICI (Mean ± SD) and Interaction	Antifungal Agent			
		Voriconazole	Posaconazole	Amphotericin B	Caspofungin
*A. fumigatus* (AF293)	FICI	0.542 ± 0.036	0.854 ± 0.253	0.729 ± 0.289	0.563 ± 0.000
	Interaction	Additive	Additive	Additive	Additive
*A. fumigatus* (clinical strain)	FICI Interaction	0.833 ± 0.144	0.583 ± 0.144	0.917 ± 0.144	0.917 ± 0.144
		Additive	Additive	Additive	Additive
*A. flavus*	FICI Interaction	0.625 ± 0.108	0.583 ± 0.036	0.667 ± 0.072	0.833 ± 0.144
		Additive	Additive	Additive	Additive
*A. niger*	FICI Interaction	0.542 ± 0.144	0.833 ± 0.144	0.75 ± 0.25	0.917 ± 0.144
		Additive	Additive	Additive	Additive
*A. terreus*	FICI Interaction	0.552 ± 0.018	0.958 ± 0.473	0.708 ± 0.072	0.833 ± 0.144
		Additive	Additive	Additive	Additive

Note: This assay was performed in triplicate. FICI: fractional inhibitory concentration index.

**Table 3:** The antifungal activity of 4E4 in combination with antifungal agents against different *Aspergillus* species.

Species	FICI (Mean ± SD) and Interaction	Antifungal Agent			
		Voriconazole	Posaconazole	Amphotericin B	Caspofungin
*A. fumigatus* (AF293)	FICI	0.729 ± 0.289	0.854 ± 0.253	0.729 ± 0.289	0.625 ± 0.108
	Interaction	Additive	Additive	Additive	Additive
*A. fumigatus* (clinical strain)	FICI Interaction	0.833 ± 0.144	0.917 ± 0.144	0.917 ± 0.144	0.854 ± 0.180
		Additive	Additive	Additive	Additive
*A. flavus*	FICI Interaction	1.167 ± 0.289	1.250 ± 0.250	1.083 ± 0.382	0.521 ± 0.130
		No interaction	No interaction	No interaction	Additive
*A. niger*	FICI Interaction	1.333 ± 0.289	1.500 ± 0.500	1.667 ± 0.289	0.792 ± 0.191
		No interaction	No interaction	No interaction	Additive
*A. terreus*	FICI Interaction	1.417 ± 0.144	1.583 ± 0.382	1.250 ± 0.250	0.771 ± 0.219
		No interaction	No interaction	No interaction	Additive

Note: This assay was performed in triplicate. FICI: fractional inhibitory concentration index.

## Data Availability

All data generated or analysed during this study are included in this published article.

## Abbreviations

The following abbreviations are used in this manuscript:

Abbreviation	Full name
A405	Absorbance at 405 nm
AMB	Amphotericin B
ATCC	American Type Culture Collection
CAS	Caspofungin
CFU	Colony-forming unit
CWFs	Cell wall fragments
DMSO	Dimethyl sulfoxide
ELISA	Enzyme-linked immunosorbent assay
EUCAST	European Committee on Antimicrobial Susceptibility Testing
FIC	Fractional inhibitory concentration
FICI	Fractional inhibitory concentration index
IA	Invasive aspergillosis
IgM	Immunoglobulin M
mAb	Monoclonal antibody
MEC	Minimum effective concentration
MIC	Minimum inhibitory concentration
MOPS	3-(N-morpholino) propanesulfonic acid
PBST	Phosphate-buffered saline containing Tween 20
PBS	Phosphate-buffered saline
POS	Posaconazole
RPMI	Roswell Park Memorial Institute
RPMI-1640-MOPS	RPMI-1640 medium buffered with MOPS
SD	Standard deviation
SDA	Sabouraud dextrose agar
SDB	Sabouraud dextrose broth
VRZ	Voriconazole
